# Relationship between workplace spirituality with organization-based self-esteem and workplace deviant behaviors among Iranian nurses

**DOI:** 10.1186/s12912-024-01908-x

**Published:** 2024-04-23

**Authors:** Behzad Sirousi Moez, Amir Sadeghi, Leili Tapak, Zahra Purfarzad

**Affiliations:** 1 Student Research Committee, School of Nursing and Midwifery, Hamadan University of Medical Sciences, Hamadan, Iran; 2 Department of Nursing, School of Nursing and Midwifery, Hamadan University of Medical Sciences, Hamadan, Iran; 3 Department of Biostatistics, School of Public Health and Modeling of Noncommunicable Diseases Research Center, Hamadan University of Medical Sciences, Hamadan, Iran; 4 Department of Nursing, Malayer School of Nursing, Chronic Diseases (Home Care) Research Center, Hamadan University of Medical Sciences, Hamadan, Iran

**Keywords:** Workplace spirituality, Organization-based self-esteem, Workplace deviant behaviors

## Abstract

**Background:**

Despite the numerous studies conducted on workplace spirituality, there is still lack of studies that have explored the relationship between workplace spirituality with organization-based self-esteem and workplace deviant behaviors. This study aims to examine the relationship between workplace spirituality with organization-based self-esteem and workplace deviant behaviors among Iranian nurses.

**Methods:**

236 nurses from 5 hospitals participated in this descriptive, analytical, and cross-sectional study from August to December 2022. Data was gathered by four questionnaires: demographic information, workplace spirituality, organization-based self-steam, and workplace deviant behaviors. The data were analyzed by SPSS 26 based on descriptive and inferential statistics (Independent Two-sample t Test, Pearson correlation coefficient and multiple regression).

**Results:**

Based on the findings, nurses had a moderate level of perception of workplace spirituality and organization-based self-esteem while having a low level of perception regarding the occurrence of workplace deviate behaviors. Results of Pearson correlation coefficient test showed a positive and statistically significant relationship between workplace spirituality and organization-based self-esteem. Additionally, there was an inverse and significant relationship between workplace spirituality and organization-based self-esteem with workplace deviant behaviors. Results of multiple regression analyses indicate that by controlling the demographic characteristics of nurses, the meaningful work and sense of community have a significant relationship with organization-based self-esteem. Furthermore, by controlling the demographic characteristics of nurses, permanent employment status, sense of community, alignment with the organization’s values, and organization-based self-esteem have a significant relationship with workplace deviant behavior.

**Conclusions:**

The study suggests that organizations must prioritize promoting workplace spirituality and organization-based self-esteem to ensure a healthy work environment and prevent workplace deviant behaviors.

## Introduction

Workplace deviant behavior is a common problem among employees in work organizations. Workplace deviant behavior refers to “voluntary behavior that violates significant organizational norms and in so doing threatens the wellbeing of an organization, its members, or both” [1]. Examples of such behavior include absenteeism, tardiness, bribery, theft, wasting the organization’s resources, sexual harassment, rumor-spreading [2, 3]. Unfortunately, workplace deviant behaviors are a common and costly challenge in healthcare centers and organizations, just like in other organizations [4]. Nurses, under stressful working conditions, are susceptible to workplace deviant behaviors due to reasons such as staff shortages, heavy workload, unclear duties, and inadequate equipment. However, limited studies have been conducted in this area among nurses [5].

One of the factors that determine workplace deviant behavior is workplace spirituality. Deviant behavior in the workplace reduces when a person has a good spiritual experience at work [6]. The employees who have a sense of spirituality in the workplace tend to be more satisfied with their jobs and less likely to engage in unethical behavior. Additionally, individuals who have a spiritual inclination tend to exhibit ethical behavior in their workplace. This is because they tend to work better in teams, show greater kindness and fairness, are more aware of others’ needs, and demonstrate traits such as honesty and trust [7]. The social control theory suggests that people are less likely to participate in deviant behavior at work if they have strong connections with social institutions like co-workers and religion. These bonds can be broken down into four elements: attachment, commitment, involvement, and belief in wider social values [8]. When employees do not experience spirituality in the workplace, they may feel angry, resentful, or dissatisfied and target their organization or colleagues with negative work behaviors [9]. According to the social exchange theory, workplace deviant behaviors can be seen as the result of an unfavorable social exchange between employees and their organization [10].

In order to establish workplace spirituality, it is imperative that employees not only comprehend the meaning and purpose of their work, but also experience a sense of solidarity and connection with their colleagues. Additionally, the values shared by employees must align with the organization’s values [11]. If the organization fails to meet their expectations, employees may react negatively, which is why it is crucial for organizations to consider workplace spirituality [12]. By implementing workplace spirituality, organizations can enhance employee organization-based self-esteem [13]. Organization based self-esteem is defined as, “the degree to which an individual believes him/herself to be capable, significant and worthy as an organizational member” [14]. Organizations that make their employees feel important, effective and valuable will witness a significant boost in their employees’ self-esteem levels [15]. Effective interactions are crucial for conveying an employee’s value to the organization. It is essential for employees to receive positive feedback from their colleagues to enhance their perception of their own value [16]. In this regard, the findings of study of Siriattakul et al. (2020) showed that spiritual leadership is effective on employees’ organizational self-esteem [17]. On the other hand, when employees’ organization-based self-esteem is damaged, they view themselves as less competent and valuable, which can lead to workplace deviant behavior [18, 19].

Workplace spirituality is an undoubtedly novel concept that has recently emerged in the management and business literature [20]. The positive impact of workplace spirituality cannot be underestimated as an increasing number of employees seek value, support, and meaning in their work [21]. It is, therefore, not surprising that workplace spirituality is becoming increasingly prevalent in organizations, and research is shifting towards examining and explaining the dimensions and indicators of workplace spirituality, as well as the factors that affect it or are affected by it [22]. On the other hand, obtaining sufficient knowledge about the levels of workplace deviant behaviors can enable hospital and nursing managers, as well as nurses themselves, to develop and implement intervention strategies to potentially reduce deviant behaviors in hospitals [23]. Although some studies have shown a correlation between workplace spirituality and deviant behaviors [24], the findings of the study showed that there was no significant direct correlation between spirituality and deviant behaviors [25]. Therefore, this study aimed to determine the relationship between workplace spirituality with organization-based self-esteem and workplace deviant behaviors among Iranian nurses.

## Methods

### Study design, sample and setting

A descriptive correlational and cross-sectional study was conducted in five hospitals affiliated with Hamadan University of Medical Sciences. The study population included all nurses working in these hospitals during the sampling period (from August to December 2022). The following formula was used to calculate the sample size for correlation studies:

$$ N={\left[\left({Z}_{\alpha }+{Z}_{\beta }\right)/C\right]}^{2}+3$$

$$ C=\frac{\text{ln}\left[\left(1+r\right)/\left(1-r\right)\right]}{2}$$

Based on previous studies [10], a correlation coefficient of 0.18, a power of 80%, an estimation error of 0.05%, and a 10% potential dropout rate, we have estimated a sample size of 250 nurses. We utilized a proportional stratified random sampling method to select the nurses based on the number of nurses employed in each hospital. This ensured that each center had an appropriate share of nurses in the sample. Subsequently, 250 nurses were randomly selected from each hospital’s list of nurses using a simple random sampling method and a table of random numbers. To be included in the study, nurses were required to have at least a bachelor’s degree in nursing, a minimum of one year of clinical experience, satisfaction, and willingness to participate. The exclusion criteria were a lack of willingness to cooperate or continue participation in any stage of the research and incomplete questionnaires.

### Data collection tools

#### Demographic information questionnaire

The first questionnaire collected demographic data, including age, gender, service history, type of employment, marital status, level of education, and type of work shift.

#### The scale of workplace spirituality

The second questionnaire measured workplace spirituality. The questionnaire was based on a standard tool introduced by Milliman et al. (2003) which was designed by Ashmos and Dachon (2000) [26]. The Persian version of this questionnaire was approved by Alizadeh in 2015. It comprised 20 items grouped into three components: meaningful work (6 items), sense of community (7 items), and alignment with the organization’s values (7 items) [27]. Participants rated their agreement with each statement on a 5-point Likert scale (1 for completely disagree to 5 for completely agree). In the present study, the average score of the questionnaire and its components was calculated by dividing the total score by the number of items and reported from 1 to 5. A higher average score indicates more workplace spirituality. This questionnaire’s validity and reliability have been confirmed in various studies [26–28]. The reliability of the instrument for each dimension ranges from 0.88 to 0.94 according to Milliman et al.‘s study, using Cronbach’s α [26]. Additionally, a study conducted in Iran reported the Cronbach’s alpha of the questionnaire to be 0.882 [27].. In the study conducted by Farmahini Farahani et al. on Iranian nurses, Cronbach’s alpha of the questionnaire was calculated for each subscale. The results were as follows: meaningful work (α = 0.824), sense of community (α = 0.784), and alignment with organizational values (α = 0.862) [28]. In the present study, the reliability of the questionnaire was calculated for each subscale: meaningful work (α = 0.894), sense of community (α = 0.861), and alignment with organizational values (α = 0.90).

#### Organizational-based self-esteem scale

The third questionnaire measured organization-based self-esteem using the organization-based self-esteem scale created by Pierce et al. (1989). It included 10 statements that evaluated employees’ beliefs and their value in the organization’s environment. Participants rated their agreement with each statement on a 5-point Likert scale (1 for completely disagree to 5 for completely agree). In the present study, the average total score of the questionnaire was obtained by dividing the total score by the number of items and was reported from 1 to 5. A higher average score indicates higher organization-based self-esteem [14]. The validity and reliability of this questionnaire have been confirmed in various studies [29, 30]. Pierce et al., reported the Cronbach’s alpha of the questionnaire to be 0.91 [14]. In studies conducted in Iran, Tahmasabi (2020) [29], and Nami et al. (2020) [30] reported Cronbach’s alpha values of 0.94 and 0.91, respectively, for this scale. The results of Cronbach’s alpha coefficients in the present study was reported as 0.883.

#### The workplace deviance behaviors scale

The fourth questionnaire was used to measure workplace deviant behaviors. The questionnaire was designed by Robinson and Bennett (1995) and included 19 items that measured two components of deviant behaviors: interpersonal deviant behaviors (7 items) and organizational deviant behaviors (12 items) [1]. Participants indicated how often they observed behaviors that were harmful to the organization or other employees at their workplace. The answers were on a 7-point scale from 1 (never) to 7 (every day). The average score of this questionnaire ranges from 1 to 7. A higher mean score indicates a higher deviant behavior in the workplace. Based on the obtained score, the behaviors were classified into three levels: 1–3 for low, 3–5 for moderate, and 5–7 for high [5]. The validity and reliability of this questionnaire have been confirmed in various studies [4, 5, 23]. Hashish (2020) calculated Cronbach’s alpha coefficients to check the internal reliability of the tool. For interpersonal deviant behaviors, the alpha coefficient was 0.973, and for organizational deviant behaviors, it was 0.980 [5]. Babamiri et al. (2022) reported the value of Cronbach’s alpha for the two components of interpersonal and organizational deviant behaviors, equal to 0.91 and 0.93, respectively [4]. Kake Mam et al. (2021) reported Cronbach’s alpha coefficients in their study for the components of interpersonal and organizational deviant behaviors, respectively, 0.93 and 0.91 [23]. In the present study, the reliability of the questionnaire was calculated for each subscale: interpersonal deviant behaviors (α = 0.906), and organizational deviant behaviors (α = 0.923).

### Data collection

The code of ethics IR.UMSHA.REC.1401.159 was obtained from the ethics committee of the research and technology department of Hamadan University of Medical Sciences in Iran. A written permit for field operations was obtained from the research and technology department of the university, and the educational and medical centers were visited with the introduction letter of this department. The questionnaires were provided by the researcher to the nurses in the service area in person and inside a packet. Upon explaining the study objectives and the questionnaire completion process along with assuring their information confidentiality, the person was requested to provide written informed consent only if they met the entry criteria and agreed to participate. The questionnaires were anonymous and identified for record-keeping purposes. The nurses were instructed to complete the questionnaire during their rest period in the nurses’ rest room. The questionnaires were to be submitted inside the provided packet. Nurses were given a 6-hour sample time to complete and submit the questionnaire. If they were unable to complete it, more time was given to them. A total of 250 questionnaires were distributed across five hospitals. 236 questionnaires were analyzed, while 13 were excluded due to outliers and unanswered questions. The response rate to the questionnaires was 94.4%.

### Data analysis

The data was analyzed using statistical software SPSS version 26. Descriptive statistics were used to calculate and report central and dispersion indicators, frequency, and percentage. Inferential statistics were also performed using Independent Two-sample t Test, Pearson’s correlation coefficient, and multiple regression. The significance level of statistical analysis was considered as *p* < 0.05.

### Ethical considerations

The study adhered to ethical guidelines by obtaining approval from the hospital management beforehand, ensuring that all participants provided informed consent, maintaining anonymity and confidentiality of the questionnaires, allowing participants to withdraw at any point, and relying only on reliable sources for data collection. The present study was confirmed by the Ethics Committee at Hamadan University of Medical Sciences (IR.UMSHA.REC.1401.159).

## Results

### Characteristics of participants

The study showed that the majority of participating nurses were female (68.2%) and married (74.6%). Most of the nurses had a bachelor’s degree (86.4%), and were officially employed (59.3%). The majority of them worked rotating shifts (80.9%). The study also revealed a clear difference in the perception of workplace deviant behaviors between nurses with a master’s degree or higher and those with a bachelor’s degree (*p* = 0.035). However, there was no significant difference in the scores for workplace spirituality, organization-based self-esteem, and workplace deviant behaviors based on demographic variables such as gender, marital status, level of education, employment status, and shift worked (*p* > 0.05) (Table 1).

**Table 1:** Characteristics of participants (*N* = 236)

Characteristic	N (%)	Workplace spirituality		Organizational self-steam		Workplace deviant behaviors	
		Mean (SD)	*P-value*	Mean (SD)	*P-value*	Mean (SD)	*P-value*
Gender							
Male	75 (31.8)	3.15 (0.72)	0.136^*^	3.71 (0.55)	0.565	2.08 (0.83)	0.110^*^
Female	161 (68.2)	3 (0.76)		3.65 (0.75)		1.90 (0.81)	
Marital status							
Married	176 (74.6)	3.13 (0.70)	0.448^*^	3.69 (0.62)	0.850	1.98 (0.81)	0.526^*^
Single	60 (25.4)	3.04 (0.84)		3.70 (0.64)		1.90 (0.86)	
Level of education							
Bachelor of nursing	204 (86.4)	3.10 (0.72)	0.815^*^	3.69 (0.62)	0.870	1.91 (0.80)	0.035^*^
Master of nursing	32 (13.6)	3.13 (0.83)		3.71 (0.64)		2.24 (0.89)	
Permanent employment status							
Yes	140 (59.3)	3.18 (0.74)	0.052^*^	3.76 (0.61)	0.051^*^	2.01 (0.84)	0.269^*^
No	96 (40.7)	2.99 (0.73)		3.60 (0.64)		1.89 (0.78)	
Shift work							
Fixed	191 (80.9)	3.11 (0.76)	0.988^*^	3.69 (0.62)	0.889^*^	1.96 (0.83)	0.982^*^
Rotating	45 (19.1)	3.10 (0.63)		3.68 (0.62)		1.96 (0.76)	

^*^independent t test

The research showed that the average age of nurses who participated was 33.49 years, with a standard deviation of 5.83 years. Their work experience was an average of 9.55 years, with a standard deviation of 5.38 years. The study found that there was a significant and direct linear relationship between the variables of age and work experience and the variables of spirituality in the work environment and organization-based self-esteem (*p* < 0.01). However, Pearson’s correlation coefficients showed that there was no significant relationship between the variables of age and work experience of nurses with workplace deviant behaviors (*p* > 0.05) (Table 1).

### Descriptive statistics

According to the nurses’ perception, the average score of the total workplace spirituality was 3.11, with a standard deviation of 0.74. Among the components of this variable, the sense of community had the highest mean (3.28, with a standard deviation of 0.75), while alignment with the organization’s values had the lowest mean value (2.81, with a standard deviation of 0.87). Additionally, the average score of nurses’ organizational self-esteem was 3.69, with a standard deviation of 0.62. The mean and standard deviation of deviant behaviors in the workplace were 1.96 and 0.82, respectively. The interpersonal deviant behavior component had a higher average than the organizational deviant behavior component (Table 2).

**Table 2:** Correlations between workplace spirituality, organization-based self-esteem and workplace deviant behaviors (*n* = 236)

Variables		Mean (SD)	Workplace spirituality				organization-based self-esteem	Workplace deviant behaviors		
			Meaningful work	Sense of community	Alignment with the organization’s values	Total		Interpersonal deviant behaviors	Organizational deviant behaviors	Total
Workplace spirituality	Meaningful work	3.24 (0.87)	1							
	Sense of community	3.28 (0.75)	*r* = 0.771^**^	1						
	Alignment with the organization’s values	2.81 (0.87)	*r* = 0.599^**^	*r* = 0.710^**^	1					
	Total	3.11 (0.74)	*r* = 0.875^**^	*r* = 0.922^**^	*r* = 0.877^**^	1				
organization-based self-esteem		3.69 (0.62)	*r* = 0.476^**^	*r* = 0.501^**^	*r* = 0.412^**^	*r* = 0.517^**^	1			
Workplace deviant behaviors	Interpersonal deviant behaviors	2.27 (1.05)	*r*=-0.262^**^	*r*=-0.387^**^	*r*=-0.419^**^	*r*=-0.403^**^	*r*=-0.361^**^	1		
	Organizational deviant behaviors	1.78 (0.79)	*r*=-0.243^**^	*r*=-0.326^**^	*r*=-0.296^**^	*r*=-0.324^**^	*r*=-0.413^**^	*r* = 0.689^**^	1	
	Total	1.96 (0.82)	*r*=-0.273^**^	*r*=-0.383^**^	*r*=-0.380^**^	*r*=-0.390^**^	*r*=-0.424^**^	*r* = 0.896^**^	*r* = 0.939^**^	1
Age		33.49 (5.83)	*r* = 169^**^	*r* = 0.179^**^	0.116	*r* = 0.172^**^	*r* = 0.175^**^	*r*=-0.042	*r*=-0.025	*r*=-0.035
Work experience		9.55 (5.38)	*r* = 0.175^**^	*r* = 0.159^*^	*r* = 0.096	*r* = 0.158^*^	*r* = 0.185^**^	*r*=-0.082	*r*=-0.041	*r*=-0.064

^**^. Correlation is significant at the 0.01 level (2-tailed)

^*^. Correlation is significant at the 0.05 level (2-tailed)

### Correlations of workplace spirituality, organization-based self-esteem, and workplace deviant behaviors

Results of Pearson correlation coefficient test showed a positive and statistically significant relationship between workplace spirituality and organization-based self-esteem (*r* = 0.517, *p* < 0.001). Additionally, there was an inverse and significant relationship between workplace spirituality (*r*=-0.390, *p* < 0.001) and organization-based self-esteem (*r*=-0.424, *p* < 0.001) with workplace deviant behaviors (Table 2).

### Associations of general characteristics and components of workplace spirituality with organization-based self-esteem

Results of multiple regression analyses indicate that by controlling the demographic characteristics of nurses, the meaningful work (β = 0.207, *p* = 0.023) and sense of community (β = 0.241, *p* = 0.019) components of workplace spirituality have a significant relationship with organization-based self-esteem. The components of workplace spirituality predict 28.8% of the variance in the organization-based self-esteem while controlling demographic characteristics (Table 3).

**Table 3:** Results of multiple regression analyses

Variables		Organization-based self-esteem		Workplace deviant behaviors	
		β	p	β	p
Gender	Male vs. Female	0.024	0.686	0.083	0.164
Marital status	Single vs. Married	0.094	0.131	0.001	0.982
Permanent employment status	Yes vs. No	0.020	0.780	0.209	0.003
Shift work	Rotating vs. Fixed	0.056	0.366	-0.013	0.838
Age		0.006	0.967	0.200	0.139
Work experience		0.141	0.315	-0.272	0.057
Workplace spirituality	Meaningful work	0.207	0.023	0.179	0.053
	Sense of community	0.241	0.019	-0.244	0.020
	Alignment with the organization’s values	0.097	0.299	-0.191	0.020
Organization-based self-esteem				-0.311	< 0.001
R^2^ (adjusted R^2^)		0.288 (0.259)		0.278 (0.246)	
F (p)		10.060 (< 0.001)		8.588 (< 0.001)	
Durbin-Watson		1.857		1.893	
Tolerance		0.161–0.913		0.178–0.913	
VIF		1.095–6.209		1.095–6.237	

### Associations of general characteristics, components of workplace spirituality, and organization-based self-esteem with workplace deviant behaviors

Results of multiple regression analyses indicate that by controlling the demographic characteristics of nurses, permanent employment status (β = 0.209, *p* = 0.003), sense of community (β=-0.244, *p* = 0.020), alignment with the organization’s values (β=-0.191, *p* = 0.020), and organization-based self-esteem (β=-0.311, *p* < 0.001) have a significant relationship with workplace deviant behaviors. The components of workplace spirituality and organization-based self-esteem predict 27.8% of the variance of workplace deviant behaviors while controlling demographic characteristics (Table 3).

## Discussion

This study showed that nurses had an average level of workplace spirituality. This finding was consistent with some previous studies [28]. However, there were also studies that reported a higher level of workplace spirituality [2, 31]. The perception of workplace spirituality among nurses can vary depending on their religious, cultural, and personal beliefs. This may be the reason for the difference in the studies. In some cultures, spirituality in the work environment is linked to personal satisfaction and a sense of purpose. Nurses may see spirituality as a way to find meaning in their work, communicate with their patients more deeply, and cope with the stress and emotional demands of their jobs. In Eastern cultures, workplace spirituality can be related to the concept of harmony and balance. Nurses may see spirituality as a way to create a harmonious work environment and promote positive relationships with colleagues and patients. In religious and native cultures, spirituality in the work environment may be rooted in the relationship between man and God or nature. Nurses may see spirituality as a way to respect the natural world and promote healing through traditional practices and rituals, or they may perceive it as a way to be close to and obey God. Therefore, the understanding of spirituality in the work environment among nurses can be a phenomenon dependent on people’s lived experience and can be understood in different ways by nurses with different cultural backgrounds and personal beliefs. The findings of the study showed that the component of sense of community had the highest mean among the components of workplace spirituality, while alignment with the organization’s values had the lowest. This means that nurses gave more importance to the group aspect of workplace spirituality.

The present study found that nurses generally have a medium to high level of their organization-based self-esteem. This finding was consistent with some previous studies [32, 33]. However, some studies have reported lower levels of organization-based self-esteem compared to the present study [34]. The study also found that nurses reported low levels of workplace deviant behavior. This finding was consistent with some previous studies [2, 5, 23]. However, the use of self-report questionnaires may lead to an underestimation of the actual levels of deviant behavior. A study conducted in Pakistan evaluated deviant work behaviors of nurses by surveying their colleagues instead of the nurses themselves. The results showed that the level of deviant behavior was evaluated as average [35]. Interestingly, the study found that nurses reported a higher level of interpersonal deviant behavior compared to organizational deviant behavior. This may be due to nurses perceiving their organization as a source of support and strength. However, if nurses feel that their organization is not supportive or does not recognize their role, they may be more likely to engage in organizational deviant behavior.

The results of present study showed that there is a positive relationship between workplace spirituality and organization-based self-esteem The results are consistent with previous studies [17, 30]. There are several reasons that can explain this relationship. Firstly, workplace spirituality can provide nurses with a sense of purpose and meaning in their work. When nurses feel that their work aligns with their personal values and beliefs, it can increase their sense of worth and organization-based self-esteem. Secondly, workplace spirituality can act as a coping mechanism for stress and emotional challenges that nurses often face in their roles. When nurses feel more capable and flexible in their work environment, they are likely to experience a boost in their self-esteem. Thirdly, workplace spirituality can strengthen the sense of sociality and support among nurses, which can positively impact their self-esteem. Lastly, when nurses feel that their work is guided by moral principles, it can increase their self-esteem, as they perceive themselves as more capable and charitable individuals. In conclusion, further research and qualitative insights from nurses themselves can provide a more comprehensive understanding of the underlying mechanisms of this relationship.

The results of this study indicate that increased perception of workplace spirituality among nurses is associated with a decrease workplace deviant behaviors. The results are consistent with previous studies [2, 36], while some studies have reported no significant relationship [10, 25, 37]. Furthermore, the study also reveals that an increase in nurses’ perception of organizational self-esteem is associated with a decrease in deviant behaviors, which is in line with other studies [18, 19]. Workplace spirituality underscores moral and human-centered values, promoting a greater sense of personal responsibility among nurses. When nurses feel connected to a higher purpose or values in their work environment, deviant behaviors that conflict with their moral principles become less likely. Additionally, spirituality in the workplace contributes to nurses’ psychological well-being by creating a sense of meaning, purpose and satisfaction, which reduces the likelihood of deviant behaviors. The study suggests that cultivating a work environment that promotes spirituality can help create a positive and supportive organizational culture, leading to greater sociality, trust, and respect among nurses, and reducing the occurrence of deviant behaviors. Spirituality in the workplace also serves as a coping mechanism for nurses, enabling them to deal with stress, conflicts, and challenges in a constructive manner, rather than resorting to deviant behaviors. Qualitative studies that encourage open dialogue and reflection on how spirituality in the workplace can positively affect behavior and the overall workplace environment are crucial for further understanding this relationship among nurses.

## Limitations

The study has several limitations that could affect the validity of the results. Firstly, the sample size of nurses was limited to only five hospitals. This could significantly reduce the representativeness of the sample and limit the generalization of the findings. Additionally, the cross-sectional design of the study can limit our understanding of causality and changes over time. The use of self-reported data may also lead to response bias and affect the accuracy of the results. It’s worth noting that the negative load of deviant work behaviors may lead to bias in response, which could further affect the validity of the results. Moreover, the demographic structure of nurses was not homogenous, which could also affect the accuracy of the findings. Lastly, due to time constraints, other possible effective variables were not included, which could have a significant impact on the results.

## Conclusion

The study found that promoting workplace spirituality can control deviant work behaviors and increase organization-based self-esteem among nurses. It recommends that nursing management should take action to foster spirituality to promote positive work behaviors among nurses. While further research is needed to explore the mechanisms involved, this study provides constructive recommendations for nursing management and education to create a more positive and ethical work environment for nurses.

## Data Availability

The data that support the findings of this study are available from the corresponding author upon reasonable request.

## References

[1] Robinson SL, Bennett RJ (1995). A typology of deviant workplace behaviors: a multidimensional scaling study. Acad Manag J.

[2] Budiman B, Ardianty S, Wigati I, Baharudin B, Aziz SFA (2022). Workplace deviant behaviour of healthcare workers in islamic hospitals. Psikis: Jurnal Psikologi Islami.

[3] Usman Z, Asif M (2022). Role of workplace ostracism and self-esteem on workplace deviance. Организационная психология.

[4] Babamiri M, Heydari B, Mortezapour A, Tamadon TM (2022). Investigation of demand–control–support model and effort–reward imbalance model as predictor of counterproductive work behaviors. Saf Health Work.

[5] Hashish EAA (2020). Nurses’ perception of organizational justice and its relationship to their workplace deviance. Nurs Ethics.

[6] Astuti RJ, Maryati T, Harsono M (2020). The effect of workplace spirituality on workplace deviant behavior and employee performance: the role of job satisfaction. J Asian Finance Econ Bus.

[7] Van der Walt F, Steyn P (2019). Workplace spirituality and the ethical behaviour of project managers. SA J Industrial Psychol.

[8] Yusof RM, Yunus NKY, Adnan AAZ (2019). Examining moderating effect of industrial relations climate on workplace spirituality and counterproductive work behaviour. Int J Acad Res Acc Finance Manage Sci.

[9] Nikmanesh S, Leilidoost F, Zarjou S, Mousavi M (2019). Investigating the role of Employee Social Avoidance Effect on the relationship between job satisfaction and Anti-production Behaviors of Hashtpar Social Security Organization. J Sustainable Hum Resource Manage.

[10] Haldorai K, Kim WG, Chang HS, Li JJ (2020). Workplace spirituality as a mediator between ethical climate and workplace deviant behavior. Int J Hospitality Manage.

[11] Khaira M (2021). Workplace spirituality and the affective commitment: emotional intelligence as moderator. Hum Resource Manage Stud.

[12] Qaiser S, Abid G (2022). Psychological contract breach and happiness at work in healthcare sector: double mediation of colleague support and deviant workplace behaviour. Pakistan J Commer Social Sci (PJCSS).

[13] Iqbal Q, Hassan SH. Role of Workplace spirituality: personality traits and counterproductive workplace behaviors in Banking Sector. Int J Manage Acc Econ 2016; 3(12).

[14] Pierce JL, Gardner DG, Cummings LL, Dunham RB (1989). Organization-based self-esteem: construct definition, measurement, and validation. Acad Manag J.

[15] Sholikhah Z, Wang X, Li W (2019). The role of spiritual leadership in fostering discretionary behaviors: the mediating effect of organization based self-esteem and workplace spirituality. Int J Law Manage.

[16] Takhsha M, Barahimi N, Adelpanah A, Salehzadeh R (2020). The effect of workplace ostracism on knowledge sharing: the mediating role of organization-based self-esteem and organizational silence. J Workplace Learn.

[17] Siriattakul P, Namdej P, Wongsurawat K. Workplace spirituality and organisation based self esteem as mechanisms linking spiritual leadership with organisational citizenship behaviour: perceived organisational support as a moderator. Asian Adm Manage Rev. 2020;3(2). 10.2139/ssrn.3900531.

[18] Atiya MAG, Sliman WMM, Elsebaie SR (2021). COVID-19 stigma and nurses’ professional quality of life, self-esteem and performance. Am J Nurs Res.

[19] Shafique I, Qammar A, Kalyar MN, Ahmad B, Mushtaq A (2020). Workplace ostracism and deviant behaviour among nurses: a parallel mediation model. J Asia Bus Stud.

[20] Utami S, Sapta IKS, Verawati Y, Astakoni IMP (2021). Relationship between workplace spirituality, organizational commitment and organizational citizenship behavior. J Asian Finance Econ Bus.

[21] Sikandar A, Kalsoom U, Khan S, Shams S. Workplace Spirituality in Relation with Organizational Commitment: Bank Employees, Nurses And University Faculty In Perspective. *PalArch’s Journal of Archaeology of Egypt/Egyptology* 2021; 18(08):4718–4727. https://archives.palarch.nl/index.php/jae/article/view/9820.

[22] Lotfi Nezhad T (2016). The relationship between spirituality and organizational commitment Razi University of Kermanshah. Res Educational Leadersh Manage.

[23] Kakemam E, Torkzadeh L, Rouzbahani M, Zahedi H, Chegini Z (2021). The relationship between workplace deviant behaviors and organizational justice among nurses: a cross-sectional survey. Nurs Forum.

[24] Teimouri H, Safari A, Jahanbazi Goujani A, Babaahmadi Milani Z (2021). The impact of spirituality in the workplace on organization identity with the mediating role of organizational health and deviant behavior. J Appl Sociol.

[25] Amin S, Situngkir S, Aira DMF (2021). Minimizing workplace deviant behaviors through workplace spirituality and organizational commitment: a case study in Indonesia. J Asian Finance Econ Bus.

[26] Milliman J, Czaplewski AJ, Ferguson J (2003). Workplace spirituality and employee work attitudes: an exploratory empirical assessment. J Organizational Change Manage.

[27] Alizadeh S (2018). Relationship between spirituality in workplace as an ethical factor and team working. Ethics Sci Technol.

[28] Farmahini Farahani M, Jaberi K, Purfarzad Z. Workplace, spirituality, Compassion satisfaction, Burnout, and secondary traumatic stress: a cross-sectional study in Iranian nurses. Perspect Psychiatr Care. 2023;2023. 10.1155/2023/7685791.

[29] Tahmasbi A (2020). The relationship between job burnout and mental health: moderating role of psychological hardiness and organization-based self-esteem. J Industrial Organizational Psychol Stud.

[30] Naami A, Qanavati S, Hashemi SE (2020). The Effect of Organizational Trust and Workplace spirituality on Organizational Citizenship Behavior and Psychological Well-being: mediating role of Organization Based Self-Esteem. Int J Psycology (IPA).

[31] Hamzeian A, Jabari E (2019). The Effect of Spirituality Workplace on Work Engagement with the mediation of Will-being nurses) case study: Pasargad Hospital). Q J Nurs Manage.

[32] Adil K, Mahmood A (2023). Enhancing Family Life Quality of nurses amid COVID-19 pandemic: the mediating role of Organization-based self-esteem in the relationship between mentoring quality and family satisfaction. SAGE Open.

[33] Mazhari Moghadam H, Alavi A (2022). The relationship between organizational self-esteem and organizational health of nurses. J Family Centered Health Care.

[34] Chen MF, Ho CH, Lin CF, Chung MH, Chao WC, Chou HL, Li CK (2016). Organisation-based self‐esteem mediates the effects of social support and job satisfaction on intention to stay in nurses. J Nurs Adm Manag.

[35] Sarwar A, Naseer S, Zhong JY (2020). Effects of bullying on job insecurity and deviant behaviors in nurses: roles of resilience and support. J Nurs Adm Manag.

[36] Shaheen A, Ghayas MM (2022). Workplace Spirituality, emotions, and deviant workplace behavior. Reviews Manage Sci.

[37] Shrestha AK, Jena LK (2021). Interactive effects of workplace spirituality and psychological capital on employee negativity. Manage Labour Stud.

